# Battery health evaluation using a short random segment of constant current charging

**DOI:** 10.1016/j.isci.2022.104260

**Published:** 2022-04-12

**Authors:** Zhongwei Deng, Xiaosong Hu, Yi Xie, Le Xu, Penghua Li, Xianke Lin, Xiaolei Bian

**Affiliations:** 1 College of Mechanical and Vehicle Engineering, Chongqing University, Chongqing 400044, China; 2 College of Automation, Chongqing University of Posts and Telecommunications, Chongqing 400065, China; 3 Department of Mechanical Engineering, Ontario Tech University, ON L1G 0C5, Canada; 4 Department of Chemical Engineering, KTH Royal Institute of Technology, 114 28 Stockholm, Sweden

**Keywords:** Electrochemistry, Electrochemical energy storage, Engineering

## Abstract

Accurately evaluating the health status of lithium-ion batteries (LIBs) is significant to enhance the safety, efficiency, and economy of LIBs deployment. However, the complex degradation processes inside the battery make it a thorny challenge. Data-driven methods are widely used to resolve the problem without exploring the complex aging mechanisms; however, random and incomplete charging-discharging processes in actual applications make the existing methods fail to work. Here, we develop three data-driven methods to estimate battery state of health (SOH) using a short random charging segment (RCS). Four types of commercial LIBs (75 cells), cycled under different temperatures and discharging rates, are employed to validate the methods. Trained on a nominal cycling condition, our models can achieve high-precision SOH estimation under other different conditions. We prove that an RCS with a 10mV voltage window can obtain an average error of less than 5%, and the error plunges as the voltage window increases.

## Highlights

• A short random charging segment enables battery health evaluation
• Two features with high correlations to battery capacity are extracted
• Three typical machine learning methods are compared for battery health estimation
• The method is verified by four types of batteries cycled under different conditions

## Graphical abstract

**Figure:**
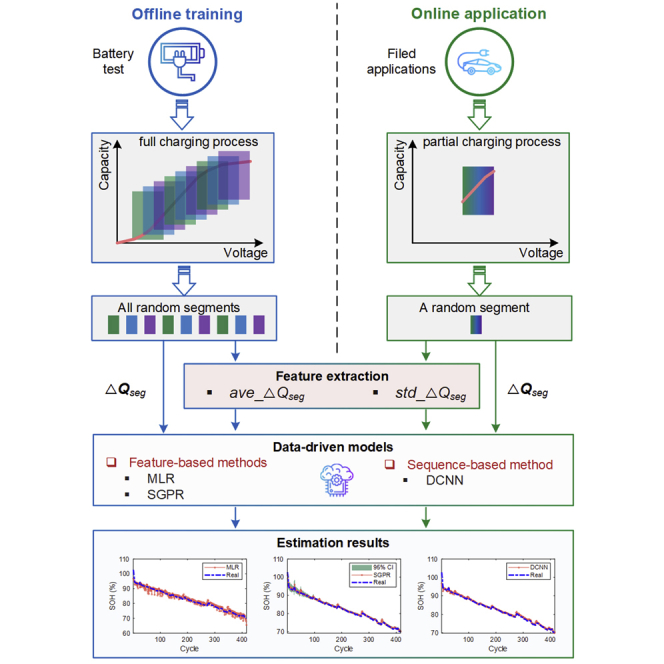

## Introduction

Lithium-ion batteries (LIBs) have made our daily lives more convenient and colorful by powering our smartphones, computers, electric vehicles, and so forth. Their advantages in energy density, power density, and long lifetime have been accelerating their penetration in various energy storage applications (Larcher and Tarascon, 2015). However, LIBs inevitably age during use and non-use time, mainly owing to the loss of lithium-ion inventory (LLI) and loss of active materials (LAM) inside the batteries (Barré et al., 2013). The direct effect of aging on battery performance is the decrease in capacity and the increase in internal resistance (Han et al., 2019). To improve the safety, efficiency, and economy of using LIBs, it is indispensable to conduct battery safety monitoring (Deng et al., 2018), residual value assessment, and timely maintenance (Hu et al., 2020), all of which heavily depend on the battery health evaluation. However, the health degradation of LIBs affected by temperature, current rate, mechanical stress, and historical operational conditions presents highly nonlinear dynamics (Attia et al., 2020; Severson et al., 2019).

From a physical point of view, the most direct method for battery health evaluation is to quantify the microscopic degradation processes of the battery, such as solid electrolyte interphase (SEI) growth (Wang et al., 2019), particle cracking (Yan et al., 2017), and lithium plating (Xiao, 2019). However, these degradation processes are coupled with each other, and their measurement is currently destructive to the battery (Reniers et al., 2019) and non-destructive techniques need to be explored. Moreover, these microscopic inspections usually require high-cost *ex situ* techniques and cannot be conducted in the field. To avoid these intractable problems, researchers developed various semi-empirical models to quantify the degradation caused by different stress factors (Schimpe et al., 2018), such as temperature, current rate, and depth of discharge. A large number of experiments are required to establish an accurate semi-empirical model, and its adaptivity to other operational conditions is still questionable. Battery electrochemical models (Doyle et al., 1993) and equivalent circuit models (Plett, 2004a) are widely used to simulate battery behaviors. As the battery health-related parameters, such as capacity and internal resistance, can be derived from the battery model, parameter identification (Rahimi-Eichi et al., 2014) and state estimation methods (Plett, 2004b) are used to obtain these parameters. However, owing to the model uncertainty and limited measurements for feedback (only voltage and temperature), it is very difficult to obtain reliable estimation results with clear physical meaning.

Recently, with the development of machine learning techniques and the availability of a large amount of high-quality battery data, various data-driven methods (Li et al., 2019; Ng et al., 2020; Shu et al., 2021) have been proposed for battery health prognostics. According to the input types, these methods can be roughly divided into two categories: feature-based methods and sequence-based methods. Feature-based methods use the extracted features as the inputs and then utilize lightweight machine learning algorithms to model the latent functions between the features and the output target, such as multiple linear regression (MLR) (Deng et al., 2021), support vector machine (Deng et al., 2016), relevance vector machine (Li et al., 2014), and Gaussian process regression (GPR) (Richardson et al., 2019; Roman et al., 2021). Various features can be extracted from the voltage, current, temperature curves during the charging/discharging process, and electrochemical impedance spectrum (Zhang et al., 2020). For example, incremental capacity (IC) and differential voltage (DV) analysis (Han et al., 2014) are two useful methods to extract features to evaluate battery health, and typical features include the peak values of the IC curves (Jiang et al., 2020; Tang et al., 2021), the valley values of the DV curves (Li et al., 2018), and the curve area within a given voltage range. In contrast, sequence-based methods directly use time-series data as the input and employ deep learning methods to achieve automatically feature extraction and nonlinear modeling, e.g., deep neural network (Roman et al., 2021; Tian et al., 2021), long short-term memory network (Deng et al., 2022b; Li et al., 2020), deep convolutional neural network (DCNN) (Shen et al., 2020), and their variants. These techniques usually use time-series data of battery current, temperature, voltage, and accumulated charge under complete or partial charging/discharging conditions as the input. Many studies have shown that both feature-based and sequence-based methods can achieve outstanding performance under specific conditions.

In many applications, the battery discharge process is a bit dynamic, while the charging process is relatively stable and usually pre-defined, such as in electric vehicles and smartphones. Therefore, many researchers developed health evaluation models based on the charging data (Jiang et al., 2020; Li et al., 2018; Shen et al., 2020; Tian et al., 2021). In these studies, a specific voltage range and a fixed start/endpoint are required to ensure that the curves of different cycles have the same reference points. However, in practical applications, the charging behaviors of users are random (Zhao et al., 2021), which means the charging start and endpoints are not fixed, and a complete charging process is very difficult to capture (Deng et al., 2022a). Furthermore, for series battery packs, the inconsistency between battery cells causes them to have different charging voltage curves and a narrow voltage overlap window (Tian et al., 2020), especially for aged cells, which hinders the health evaluation of each cell. In short, it is still a significant challenge to conduct battery health evaluation based on a random and short charging segment in real applications.

**Figure 1:**
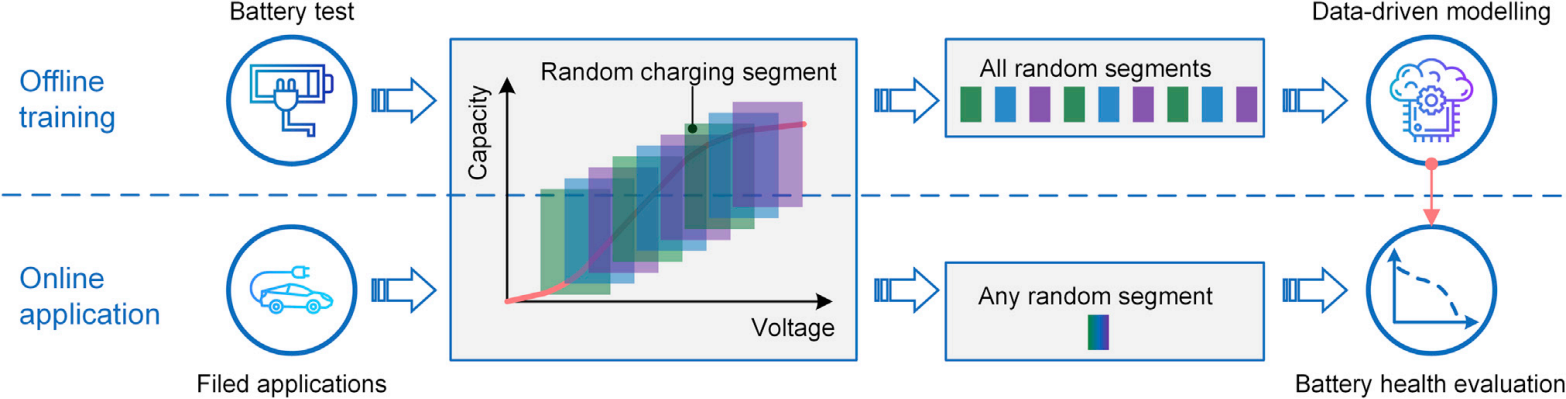
Schematic of the proposed method

In this work, we develop data-driven methods to accurately estimate battery state of health (SOH) using a random charging segment (RCS) extracted from the constant current process. The proposed methods are validated with four types of commercial batteries (75 cells in total) cycling under different temperatures and discharging rates. As schematically shown in Figure 1, we first divide the constant current (CC) charging curve into dozens of segments and extract a capacity increment sequence in each segment. We show that the capacity increment sequence evolves in a certain pattern as the battery ages, and its average value and SD have high correlations with the battery SOH. Then, we analyze the correlations under different numbers of segments and find that even a short segment is highly correlated with battery SOH. Finally, two types of machine learning algorithms (features-based and sequence-based) are used to model the SOH estimators. In the training process of the data-driven models, all RCSs are used as input, but only an RCS is required as input for the online application. We prove that the developed methods can achieve high accuracy using even an RCS with an extremely small voltage window (i.e., 10mV).

## Results

### Charge capacity evolution

**Figure 2:**
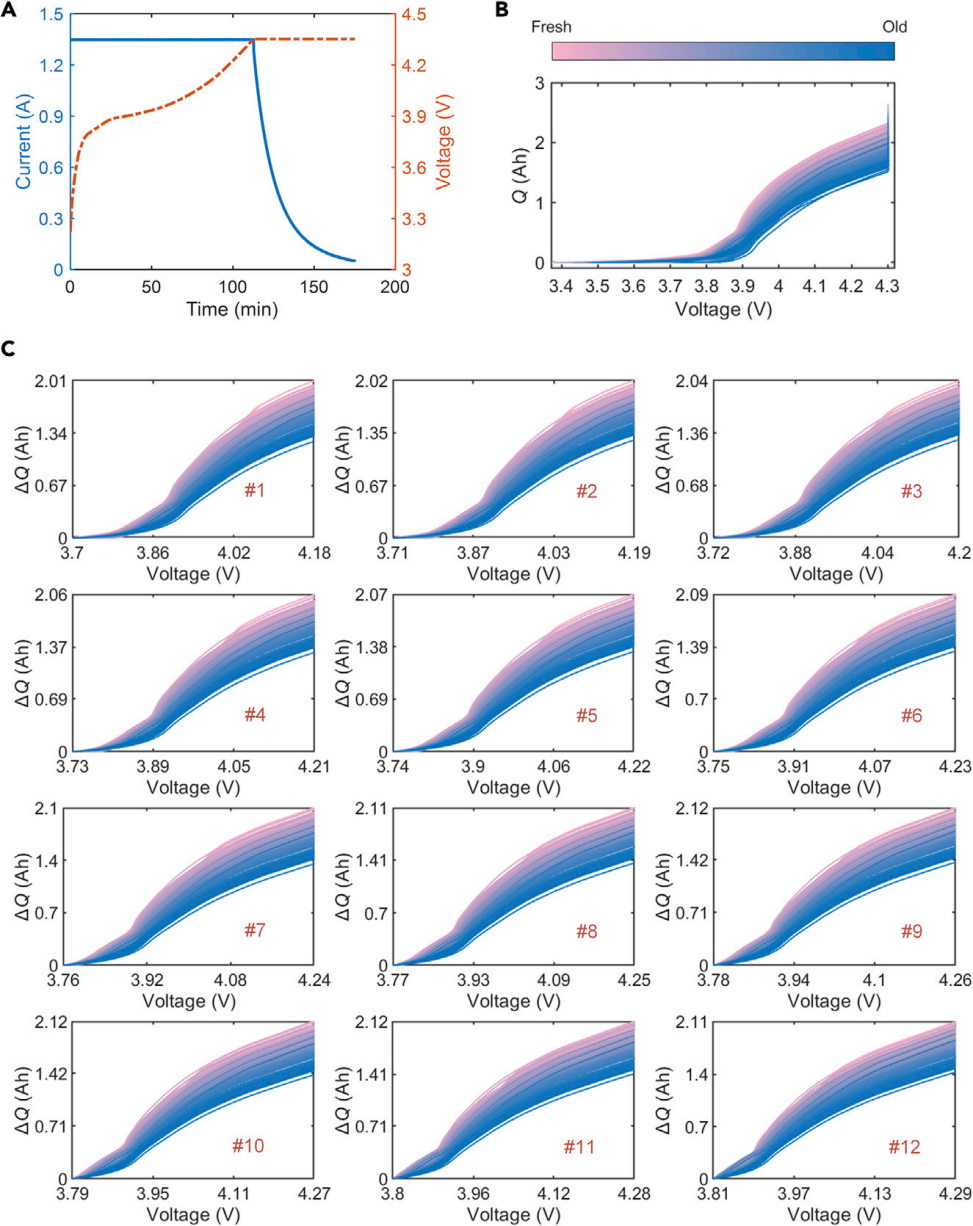
Charge capacity evolution as battery ages An NMC-LCO cell is taken as an example. (A) CC-CV charging policy. (B) Battery charge capacity curves as a function of voltage at different aging levels. (C) Capacity increment sequences in different voltage segments. The charge capacity sequence corresponding to the voltage range of 3.70–4.29V is divided into 12 segments, and each segment is denoted by a symbol #*x* (1 ≤ *x* ≤ 12).

Taking the data of a LiNi_x_Mn_y_Co_1−x−y_O_2_ blended with LiCoO_2_ (NMC-LCO) cell as an example, we investigate the evolution of battery charge capacity with the number of cycles. Figure 2A and Figure 2B show the constant current-constant voltage (CC-CV) charging profiles and the corresponding charge capacity (***Q***) as a function of voltage, where the color denotes the number of cycles. It shows that the capacity curves gradually shift downward as the battery ages. Table 3 presents parameters setting used to extract the ***Q*** from different batteries. Furthermore, we divide the voltage range of 3.70–4.29V into 12 segments according to (Equation 2) and extract the capacity increment sequences (▵***Q***_*seg*_) from the segments (Figure 2C). After this segmentation, each segment has a 0.48V voltage window. Some patterns in the evolution of charge capacity as the battery ages can also be observed in these partial charging segments.

**Table 3:** Parameter settings for capacity increment sequence extraction

Battery types	*V*_*start*_ (V)	*V*_*end*_ (V)	▵*V* (V)	*n*
NMC-LCO	3.7	4.29	0.01	60
NCA/NMC	3.6	4.19	0.01	60
LFP	3.0	3.59	0.01	60

### Correlation analysis

**Figure 3:**
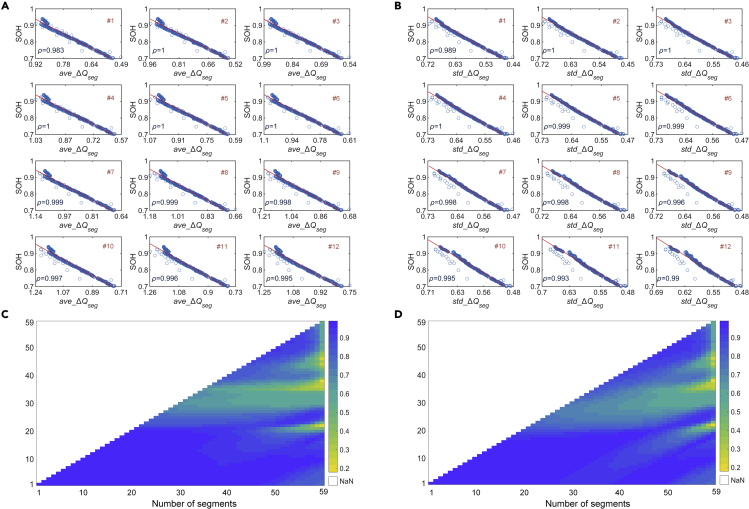
Correlation analysis of extracted features for NMC-LCO battery The charge capacity sequence corresponding to the voltage range of 3.70–4.29V is divided into *m* segments (▵*Q*_*seg*_). The *ρ* between the features and battery SOH are analyzed. (A) Correlation of *ave*_▵*Q*_*seg*_ for each segment when *m* is equal to 12. (B) Correlation of *std*_▵*Q*_*seg*_ for each segment when *m* is equal to 12. (C) Correlation of *ave*_▵*Q*_*seg*_ for each segment as *m* varies from 1 to 59. (D) Correlation of *std*_▵*Q*_*seg*_ for each segment as *m* varies from 1 to 59.

To evaluate the usefulness of the partial capacity segments for the battery health evaluation, we calculate two statistical characteristics of ▵***Q***_*seg*_ and analyze the correlations (*ρ*) between them and battery SOH. We choose the average value of ▵***Q***_*seg*_ (*ave*_▵*Q*_*seg*_) and the SD of ▵***Q***_*seg*_ (*std*_▵*Q*_*seg*_) as features, and their correlations with the SOH of NMC-LCO battery are shown in Figure 3. The correlations when the voltage range is divided into 12 segments are illustrated in Figures 3A and 3B. It can be observed that the two features have a *ρ* value close to one for almost all segments. To analyze the effect of the number of segments on the correlations, we further calculate the correlations of different segments under various segmentation operations, and the results are shown in Figures 3C and 3D. According to (Equation 2), we know that a smaller voltage window can be obtained with a larger number of segments. From the two heatmaps, it is clear that a high correlation (>0.8) can be maintained for all segments until the number of segments exceeds 30, and the first 20 segments always have high correlations no matter how many segments are defined. We also analyze the correlations of features for the other three types of batteries (see Figures S1–S3).

**Figure 4:**
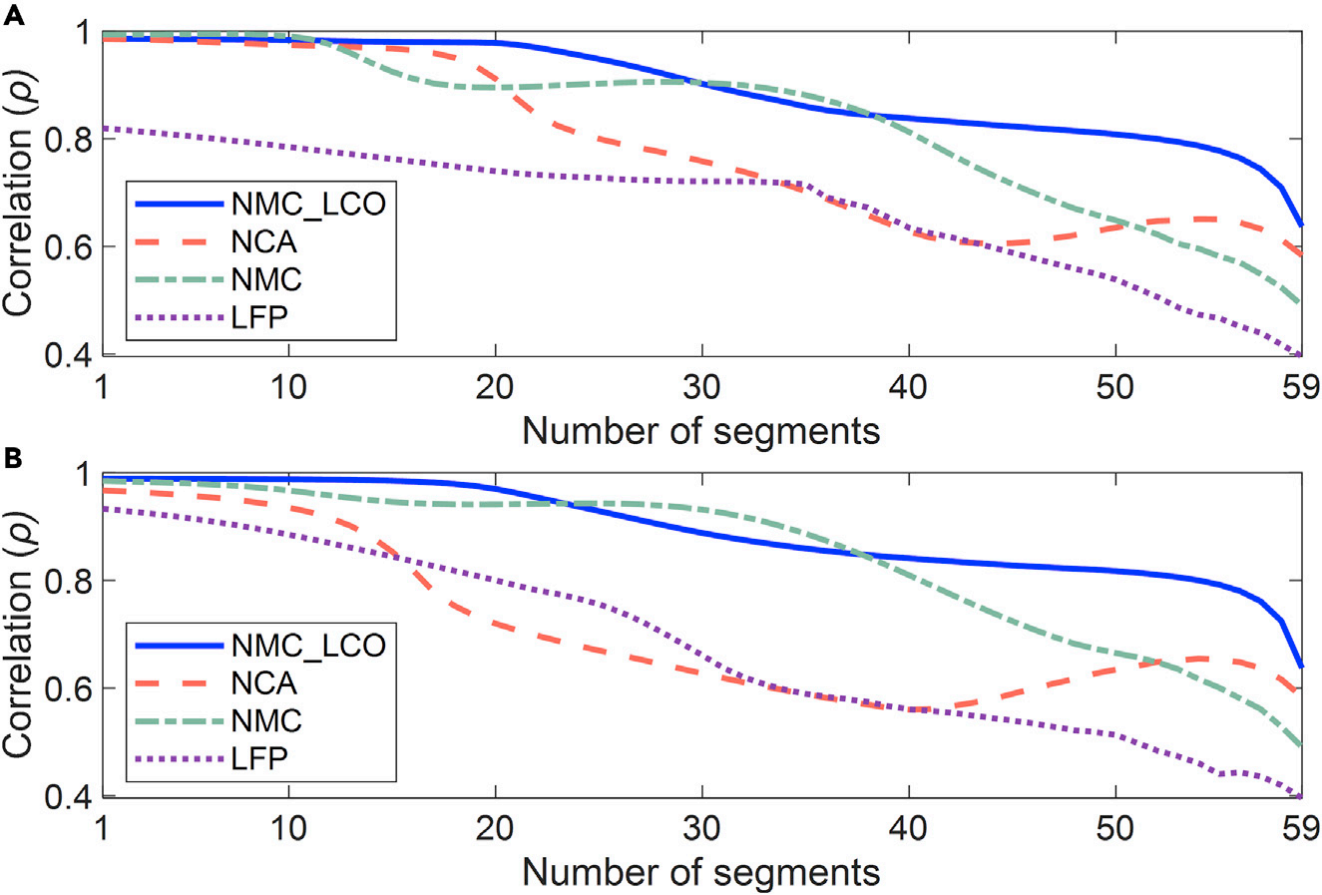
Comparison of feature correlations for different batteries The capacity sequence is divided into *m* segments, and *m* varies from 1 to 59. For each segmentation operation, the AACs of features for different segments are calculated. (A) AACs of *ave*_▵*Q*_*seg*_. (B) AACs of *std*_▵*Q*_*seg*_.

For each segmenting operation, we calculate the average absolute correlation (AAC) of each feature for different segments. Figure 4 compares the variation of the AACs with the number of segments for different batteries. All the batteries are cycling in a 25°C chamber with the same charging policy (0.5C CC-CV). For the discharging policy, the NMC-LCO battery is discharged at 1.5 CC, while the other three types are discharged at 0.5 CC. Owing to the difference in electrochemistry, the correlations of the four batteries present different patterns of change. In contrast, for the same battery chemistry, the correlation variation of its two features is highly consistent. The correlation generally decreases as the number of segments increases, except for a partial recovery in NCA battery. For all types of batteries, high correlations of the two features with the battery SOH appear when the number of segments is less than 20. Even when the number of segments is 59, which corresponds to a voltage window of 10mV, a correlation over 0.5 is obtained for NMC-LCO, NCA, and NMC batteries, and around 0.4 for LFP battery. Compared with other battery types, it is more difficult for LFP battery to extract features highly related to battery SOH based on the ▵***Q***_*seg*_, which increases the difficulty of its SOH estimation.

### Battery state of health estimation

We use two types of data-driven methods to model the battery SOH estimator. The first one is the feature-based method, which uses selected features as input. By comparison, the second one, i.e., the sequence-based method, can directly use the raw data sequence as input.

In this study, one simple algorithm, (MLR) and two state-of-the-art machine learning algorithms (sparse GPR and DCNN) corresponding to the above-mentioned two types of methods are employed to construct battery SOH estimators. The MLR is a typical method to model the linear relationship between input features and output target. In general, the better the linear correlation between the features and the output, the higher the accuracy of MLR estimation. The sparse GPR (SGPR) can efficiently capture the nonlinear relationships between the inputs features and output and provide a probabilistic prediction of the target. For the DCNN, it can infinitely approximate the nonlinear characteristics of the process owing to its deep learning mechanism. In this SOH estimation problem, the MLR and SGPR take the two features (*ave*_▵*Q*_*seg*_ and *std*_▵*Q*_*seg*_) and the mean value of the corresponding voltage sequence as inputs, while the DCNN uses the capacity increment sequence (▵***Q***_*seg*_) and the corresponding voltage sequence as inputs directly. We convert the high dimensional sequences into images as input to the DCNN, which can automatically extract features from the front layers of the network.

As illustrated in Figure 1, to achieve battery SOH estimation based on any RCS, the training samples need to cover all segments. This increases the sample size by dozens of times (usually up to 10,000), resulting in a regular GPR being unable to complete the training on a regular computer (computational complexity is *O*(*n*^3^), *n* is the sample size). In this regard, we use a sparse GPR and it can significantly reduce the computational burden by introducing inducing points (Candela and Rasmussen, 2005).

**Figure 5:**
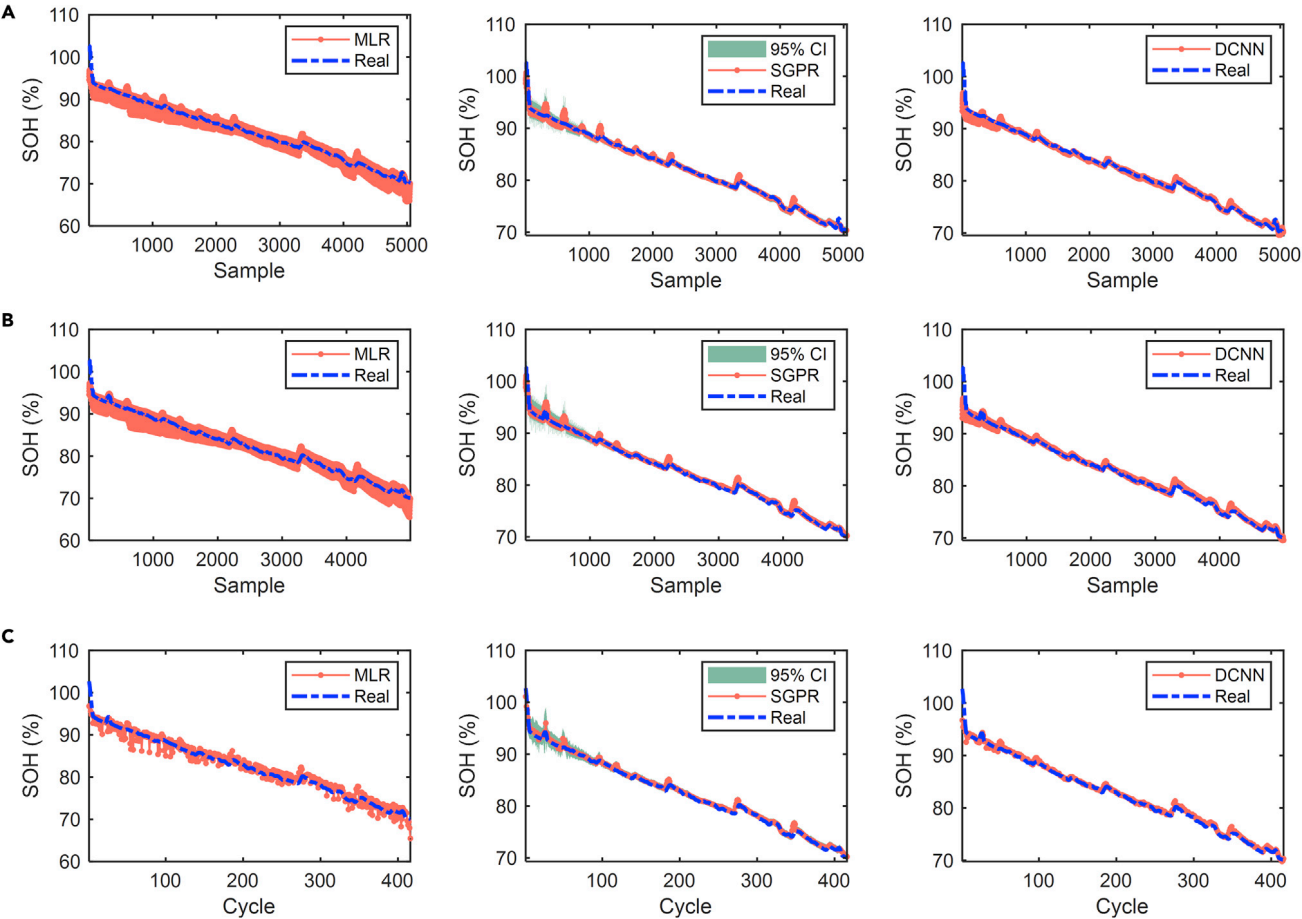
SOH estimation results of NMC-LCO batteries The capacity sequence is divided into 12 segments. MLR, SGPR, and DCNN methods are used to estimate battery SOH. The Dash line represents actual values and the dashed-dotted line represents the estimated values. (A) Training results. (B) Test results using all segments for each cycle. (C) Test results using a random segment for each cycle.

The SOH estimation results for NMC-LCO batteries are presented in Figure 5, including the training process, the test process using all segments, and the test process using a random segment for each cycle. The results of the other three types of batteries are shown in Figures S4–S6. The statistical errors of SOH estimation for the four types of batteries are summarized in Table 1. All the mentioned methods are trained on a cell under 25°C, 0.5C CC-CV charging and 0.5C discharging conditions (except for 1.5C for NMC-LCO), and are tested on another cell with the same battery type and cycling condition. We define the above cycling condition as a nominal cycling condition. In the modeling processes, we also divide the voltage range into 12 segments, which means that 12 samples are generated per cycle. Therefore, the *x*-axis labels in the training (Figure 5A) and test results (Figure 5B) are denoted by “sample” rather than “cycle.” To mimic the random charging behaviors of users, we perform uniformly distributed sampling and select one from 12 segments for each cycle as the final estimated value of the cycle (Figure 5C). It is worth noting that for the SGPR and DCNN methods, the model obtained after each training is different owing to the random setting of initial parameters. To obtain reliable results, we run 20 times of training and test processes, and take their average values as the final results.

**Table 1:** Statistical errors of SOH estimation for different batteries

	Errors	NMC-LCO			NCA			NMC			LFP		
		MLR	SGPR	DCNN	MLR	SGPR	DCNN	MLR	SGPR	DCNN	MLR	SGPR	DCNN
Training	MAE (%)	0.97	0.29	0.32	1.80	0.31	0.32	0.89	0.80	0.31	1.40	0.20	0.23
	RMSE (%)	1.28	0.52	0.66	2.29	0.63	0.52	1.35	1.40	0.46	1.82	0.70	0.41
Test	MAE (%)	1.02	0.30	0.37	1.88	1.97	0.95	0.84	0.83	0.40	1.30	0.34	0.27
	RMSE (%)	1.30	0.50	0.68	2.48	2.07	1.18	1.38	1.51	0.54	1.74	0.81	0.49
Test (random)	MAE (%)	1.05	0.29	0.36	1.93	1.96	0.95	0.91	0.91	0.39	1.31	0.35	0.27
	RMSE (%)	1.33	0.47	0.63	2.55	2.07	1.18	1.46	1.60	0.53	1.72	0.83	0.47

The above results show that all the methods can obtain high-precision SOH estimation for different battery chemistries, with a mean absolute error (MAE) lower than 2% and a root-mean-square error (RMSE) lower than 2.5% in the test process. The DCNN achieves the best performance, and its MAEs and RMSEs are lower than 1 and 1.2%, respectively. Owing to the inability to model nonlinear characteristics, the estimation accuracy of the MLR is always lower than the SGPR and DCNN. Besides, we can find that the estimation errors based on random segments are almost the same as that of using all test segments. This is because of the use of uniformly distributed sampling. According to the above results, we demonstrate that a high-accuracy data-driven estimator can be built as long as the data changes in some pattern with the output, whether it is a feature-based method or a sequence-based method.

We further analyze the effect of the number of segments on the accuracy of SOH estimation. Note that with a larger number of segments, we can get a smaller voltage window for each segment. For instance, when the number is 59, the voltage window is only 10 mV. Besides, for battery health evaluation, it is of practical importance to construct a model based on a specific cell but can maintain its accuracy for other cells. Therefore, we further test the accuracy of the models on other cells of the same chemistry. The cycling conditions of the four types of batteries are explained in Table 2. NMC-LCO batteries have the same cycling conditions, while NCA, NMC, and LFP have different cycling temperatures and discharging rates. The relationship between the capacity and the cycle number is shown in Figure S7 for all the batteries. This figure indicates that the rate of capacity degradation is significantly affected by temperature and discharging rates. In addition, we can see that the capacity of parts of batteries decays too fast under certain cycling conditions and reach a capacity range (area under black dashed-dotted line in Figure S7), to which the batteries under the nominal cycling condition have not decayed. To examine the accuracy of the models under severe capacity degradation, we still use the models established under nominal cycling conditions to estimate the SOH in this area.

**Table 2:** Battery information and experimental setting

Battery types	Contributors	Manufacturers	Nominal capacity (Ah)	Temperature (°C)	Charge & discharge policies	Battery Numbers
NMC-LCO	HNEI	LG Chem	2.8	25	0.5C CC-CV & 1.5C CC	14
NCA	SNL	Panasonic	3.2	15/25/35	0.5C CC-CV & 0.5C/1C/2C CC	18
NMC	SNL	LG Chem	3	15/25/35	0.5C CC-CV & 0.5C/1C/2C/3C CC	22
LFP	SNL	A123	1.1	15/25/35	0.5C CC-CV & 0.5C/1C/2C/3C CC	21

**Figure 6:**
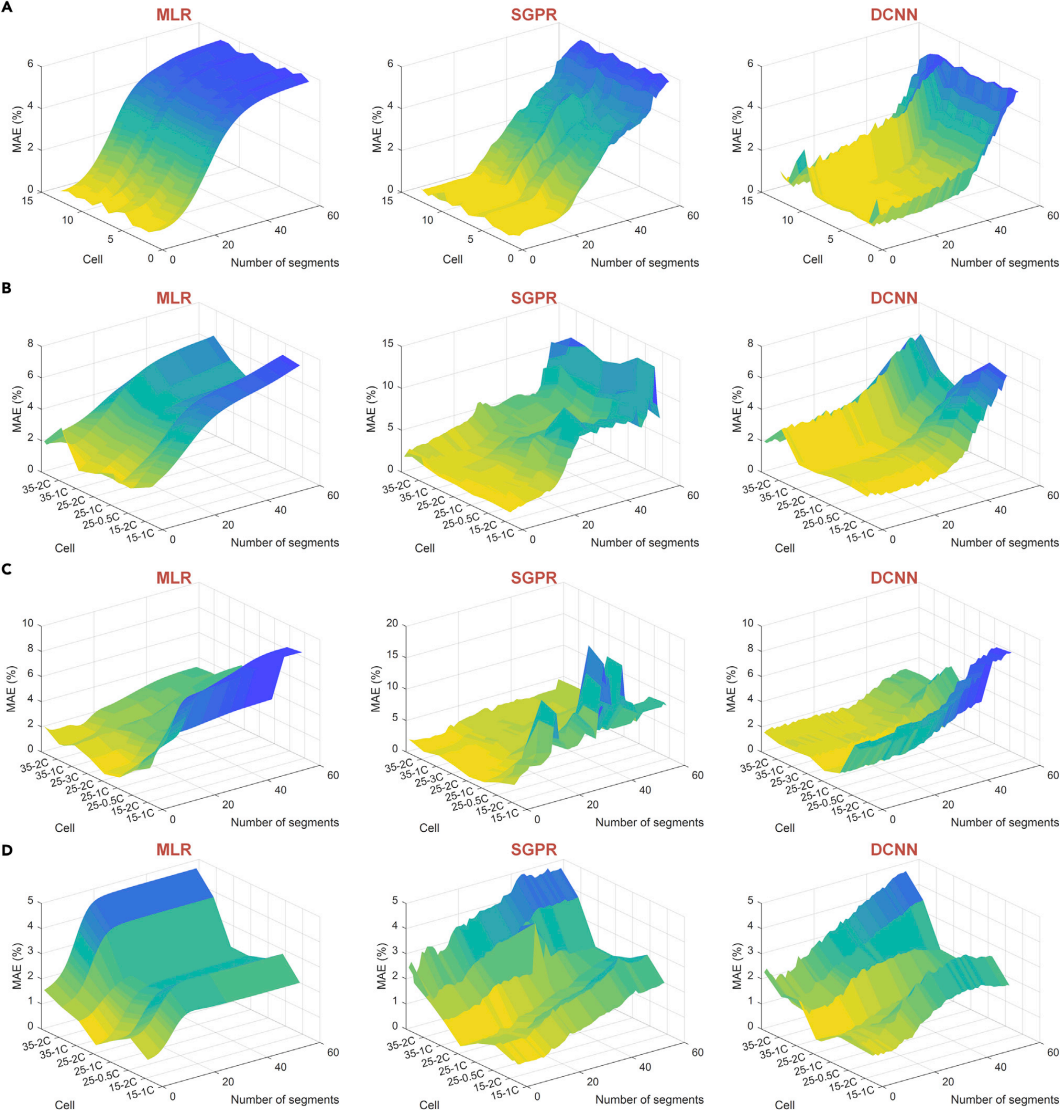
The MAEs of SOH estimation for four types of batteries Using the data of one cell to train MLR, SGPR and DCNN models, and the remaining cells are used to test the models. The variation of errors with the number of segments is also given. The symbol “T1-C1” in tick labels denotes the cell is cycled in a chamber with T1 temperature and C1 discharging rate. (A) NMC-LCO cells. (B) NCA cells. (C) NMC cells. (D) LFP cells.

The MAEs of SOH estimation for the four types of batteries are shown in Figure 6, in which the errors are plotted as a function of the number of segments and cycling conditions (except for NMC-LCO, which uses cell numbers). Owing to the requirement of convolutional operation in the DCNN, the length of the input sequence cannot be too small. We set the lowest limit of the length equal to five for the DCNN, thus there are at most 55 segments for each cycle.

For NMC-LCO cells, the estimation accuracy of different cells is almost the same, and the maximum MAEs of the three models are lower than 6%. Even using an extremely small voltage window (10 mV), an acceptable estimation result can still be obtained. And when a voltage window of 100 mV is used, a MAE of SOH lower than 3% can be obtained by the SGPR and the DCNN models. However, the above outstanding performance is owing to the same cycling condition between the trained and test cells.

For NCA, NMC, and LFP batteries, when several cells are cycled under the same condition, the average values of MAEs are used to indicate the results of this cycling condition. We can find that the MAE increases as the number of segments increases or the cycling conditions deviate from the nominal cycling condition. The influence of temperature on the estimation errors is different for the three types of batteries. For NMC batteries, a lower temperature increases the error; for LFP batteries, a higher temperature increases the error; while for NCA batteries, both a higher temperature and a lower temperature increases the error. In contrast, the influence of discharging rate on the estimation errors is insignificant. In addition to the metric of MAE, the RMSEs of SOH estimation for four types of batteries are also shown in Figure S8, and the same influence law from temperature can be observed.

**Figure 7:**
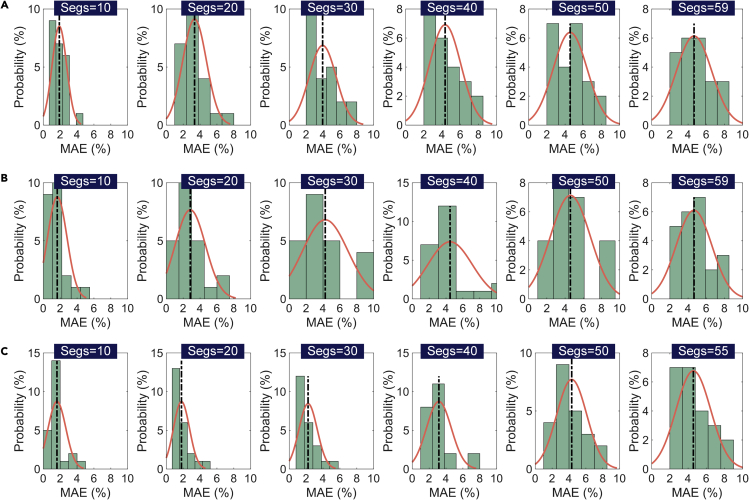
The distributions of MAEs at different numbers of segments The vertical dotted line represents the position of the mean value, and the symbol “segs” denotes the number of segments. (A) MLR method. (B) SGPR method. (C) DCNN method.

To better evaluate the robustness of the models constructed using different numbers of segments and under different cycling conditions, the distributions of MAEs at different segments are shown in Figure 7 (the distributions of RMSEs are shown in Figure S9). The distribution is calculated based on the results under the same number of segments for the four types of batteries under different cycling conditions. It can be observed that when the number of segments is less than 10, a mean MAE of less than 2% can be obtained for each method. Even if the number of segments is up to 55 or 59 (corresponds to a 10mV voltage window), a mean MAE less than 5% and a mean RMSE around 6% can be guaranteed. Note that a larger number of segments means a smaller voltage window for SOH estimation. This proves that an RCS with a 10mV voltage window can achieve an acceptable SOH estimation. Meanwhile, we can observe that a sequence with a big voltage window can capture more battery degradation information and has better generalization for different working conditions. However, it is difficult to obtain a big voltage window in the charging process in many applications. Therefore, we have to sacrifice some accuracy to ensure the availability of the models.

## Discussion

In this article, we develop data-driven models to achieve accurate battery health evaluation using an RCS extracted from the constant current charging process. We prove that capacity increment sequences in the CC charging process are informative for battery health evaluation. Two features extracted from these sequences are closely related to battery SOH. Two feature-based methods (i.e., the MLR and SGPR) and one sequence-based method (i.e., the DCNN) are used to construct data-driven models for SOH estimation. The proposed models are trained using the data of a cell under the nominal cycling condition and subsequently tested on other cells with the same or different conditions (difference in temperature and discharging current rates). The developed methods are validated using four types of batteries (75 cells in total), with the error lower than 2% when the voltage window is up to 500mV. Moreover, an average estimation error lower than 5% can be obtained even when the voltage window is less than 10mV. Our work substantiates that it is promising to use an RCS with a narrow voltage window to achieve accurate health evaluation for LIBs. As only the short random charging data is needed, the proposed methods can be applied to a variety of scenarios.

### Limitations of the study

In this article, the proposed methodology is verified under cycling conditions with constant ambient temperatures, constant discharging currents, and full charge and discharge. It is valuable to conduct more verifications under other cycling conditions, such as changing ambient temperature, dynamic discharge, and shallow charge and discharge. These will be investigated in our future work.

## STAR★Methods

### Key resources table

**Table:** 

REAGENT or RESOURCE	SOURCE	IDENTIFIER
**Deposited data**		

Battery Archive	The battery datasets are from Hawaii Natural Energy Institute (HNEI) and Sandia National Laboratories (SNL)	https://www.batteryarchive.org/index.html

**Software and algorithms**		

MATLAB R2018a	MathWorks	https://www.mathworks.com
GPML Matlab Code version 4.2	The code is written by Carl Edward Rasmussen and Hannes Nickisch	http://www.gaussianprocess.org/gpml/code/matlab/doc/

### Resource availability

#### Lead contact

Further requests for information should be directed and will be handled by the corresponding author and lead contact, Zhongwei Deng (dengzhongw@cqu.edu.cn).

#### Material availability

This study did not generate new materials.

### Method details

#### Dataset description

The cell cycling dataset of four different types of batteries is used in this work, and all of them come from an open-source battery data website. The information of batteries and experimental settings are listed in Table 2. All the batteries use graphite material as their negative electrodes, but material of their electrolytes are not disclosed. The battery cells are divided into different types based on the positive materials, namely, LiNi_x_Co_y_Al_1−x−y_O_2_ (NCA), LiNi_x_Mn_y_Co_1−x−y_O_2_ (NMC), LiFePO_4_ (LFP), and a blend of NMC and LiCoO_2_ (NMC-LCO). The dataset of NMC-LCO is contributed by Hawaii Natural Energy Institute (HNEI) (Devie et al., 2018), and the other three are contributed by Sandia National Laboratories (SNL) (Preger et al., 2020). All the battery cells are cycled under the same constant current-constant voltage (CC-CV) charging profile (0.5C CC-CV), but different CC discharging profiles with different current rates (0.5C, 1.5C, 1C, 2C, and 3C). Although the dataset of SNL including cells cycled under different depths of discharge (DODs), only the data of 100% DOD is used for this study. The benchmark SOH of the cell is calculated by,

(Equation 1) $$\mathrm{SO}H_{i}=\frac{C_{i}}{C_{n}}$$

where *C*_*n*_ is the nominal capacity of the battery, and *C*_*i*_ is the actual capacity at *i*th cycle, which is equal to the maximum discharge capacity under CC discharging.

#### Capacity increment sequence extraction

In a battery charging process, battery charge capacity can be calculated by integrating battery current over time. For constant current (CC) charging, the battery voltage is usually monotonically increasing (or filtered to maintain monotonicity). Given a constant voltage range [*V*_*start*_, *V*_*end*_] and a fixed voltage interval ▵*V*, a charge capacity sequence (***Q*** = [*Q*_*1*_, *Q*_*1*_, …, *Q*_*n*_]) can be extracted by interpolating the charge capacity curve with respect to the voltage sequence, where *n* = (*V*_*start*_ – *V*_*end*_)/▵*V*+1 (Deng et al., 2021). Considering the difference in voltage platforms for the four types of batteries, different parameters setting is used to extract the ***Q*** from different batteries and are listed in Table 3. The *V*_*start*_ is set to a voltage point corresponding to about 5% SOC of the battery because 5% SOC is usually reserved to avoid battery over-discharge in practical applications. The *V*_*end*_ is set to a value slightly away from the charging cut-off voltage to avoid the influence of data fluctuations when switching to the constant voltage (CV) charging stage.

To achieve accurate battery SOH estimation based on partial charging data, the charge capacity sequence can be further divided into dozens of segments (***Q***_*seg*_). Given a fixed length (*h*) of the segment and a stride size (*s*), *m* segments can be extracted,

(Equation 2) $$m=\mathrm{floor}\left(\frac{n-h}{s}\right)+1$$

where the function *floor*(.) gets the largest integer no more than the input value. In this study, *s* is set to 1, corresponding to one ▵*V* voltage interval. As listed in Table 3, a charge capacity sequence, ***Q*** = [*Q*_*1*_, *Q*_*1*_, …, *Q*_*n*_], can be extracted with *n* = 60 for all batteries. Setting *h* = 49, which corresponds to a 0.48V voltage window, then *m* = 12, which means ***Q*** can be divided into 12 ***Q***_*seg*_, as shown in Figure 1 for NMC-LCO battery. Due to the random charging behaviors of users, the charging start point is not fixed, thus it is impossible to calculate the exact (or absolute) charge capacity values in practical applications. To overcome this problem, the capacity sequence is replaced by the capacity increment sequence (▵***Q*** _*seg*_ = ***Q***_*seg*_ − *Q*_*seg*,1_) in each segment.

For each ▵***Q***_*seg*_, its average value (*ave*_▵*Q*_*seg*_) and standard deviation (*std*_▵*Q*_*seg*_) can be extracted as features. The Pearson correlation coefficient (*ρ*) is used to provide the strength of the linear correlation between the features and battery SOH. The correlation analysis results in four different types of batteries are illustrated in Figures 2 and S1–S3.

#### Multiple linear regression

To model the linear relationship between the input features and output target, an MLR is a commonly used method. Its expression is,

(Equation 3) $${\hat{y}}_{i}=\sum_{j=1}^{n}{\hat{w}}_{j}x_{j}+{\hat{w}}_{0}$$

where ${\hat{y}}_{i}$ is the predicted SOH, *x*_*j*_ is the input feature, *n* is the number of features, and ${\hat{w}}_{j}$ is the weight. The objective function of this regression problem is often defined to minimize the mean square error of the output. When there are many features, a regularization technique can be introduced to prevent the model from overfitting during the training process (Severson et al., 2019).

#### Gaussian process regression

To better capture the nonlinear relationship between the input features and the battery SOH, the GPR technique is employed. GPR is a machine-learning framework with non-parametric modeling and uncertainty evaluation (Williams and Rasmussen, 2006). For a typical regression problem, observations usually contain Gaussian white noises and can be modelled as,

(Equation 4) $$y_{i}=f(x_{i})+N\left(0,\sigma_{n}^{2}\right)$$

where *x*_*i*_ is the *i*th input features, $\sigma_{n}^{2}$ is the noise covariance, and ***f***(***x***)=[*f*(*x*_1_), *f*(*x*_2_), …, *f*(*x*_*n*_)] is a Gaussian process. ***f***(***x***) can be described as ***f***(***x***) ∼ *N* (0, **K**), where *K*_*ij*_=*k* (*x*_*i*_, *x*_*j*_) is the covariance kernel function, which is a measure of distance between points *x*_*i*_ and *x*_*j*_. A squared exponential kernel function is the most widely used, and is expressed as,

(Equation 5) $$k\left(x_{i},x_{j}\right)=\sigma_{f}^{2}\exp\;\left(\frac{-{\left\|x_{i}-x_{j}\right\|}^{2}}{2l^{2}}\right)$$

where *σ*_*f*_ and *l* are hyperparameters, which determine the amplitude of the kernel function and the importance of each input feature, respectively. Given training samples, the hyperparameters [*σ*_*f*_, *l, σ*_*n*_] of GPR can be optimized by maximizing the marginal likelihood. Due to the matrix inversion in solving the maximum likelihood estimation problem, the computational complexity is *O*(*n*^3^) for a regular GPR. When the size of the training space is very large, the training of the regular GPR is intractable. To overcome this problem, a sparse GPR with a specific number of inducing points is employed (Candela and Rasmussen, 2005). Only *m* training samples are selected from the original training set, thus the computational complexity can be reduced to *O*(*m*^2^*n*). In this paper, the GPR-based SOH estimation is realized by using the Gaussian processes for machine learning (GPML) toolbox (Williams and Rasmussen, 2006).

#### Deep convolutional neural network

The DCNN has been successfully used in image recognition, and the elements, lines, and shapes of a picture can be captured by different layers (Szegedy et al., 2015). Due to its ability to nonlinear modeling and automatic feature extraction (Gao and Lu, 2021), we use it to estimate battery SOH directly based on the ▵***Q***_*seg*_. In addition to the ▵***Q***_*seg*_, the corresponding voltage sequence is also used as the input of the DCNN. Unlike color images that have an input size equal to *n*×*n*×3, the above features can only form input with a size equal to *n*×2×1, thus 1D CNN is used to construct the network. In this study, the DCNN-based SOH estimation model mainly consists of two 1D convolutional layers, one maximum pooling layer, and one fully-connected layer. The structure of the developed DCNN is shown in Table S1. Since the input size is changing (*n* varies from 6 to 60 in this case), to ensure that the size of the input to the maximum pooling layer is constant, the size of the first two convolutional layers is also set to be variable. The stride size is default to one, and no padding is used. In each convolutional layer, a batch normalization technique is applied to improve the performance and stability, and a rectified linear unit (ReLU) activation function is subsequently used to learn the nonlinear relationships.

#### Evaluation criteria

Two statistical characteristics of the SOH estimation errors are chosen to evaluate the model performance. The mean absolute error (MAE), and root mean square error (RMSE) are respectively defined as,

(Equation 6) $$MAE=\frac{\sum_{i=1}^{n}\left|y_{i}-{\hat{y}}_{i}\right|}{n}$$

(Equation 7) $$RMSE=\sqrt{\frac{\sum_{i=1}^{n}{\left(y_{i}-{\hat{y}}_{i}\right)}^{2}}{n}}$$

where $y_{i}$ is the observed battery SOH, ${\hat{y}}_{i}$ is the estimated SOH, and n is the total number of samples.

## Data Availability

The raw dataset used in this study is available at https://www.batteryarchive.org. The code for data processing, features extraction and data-driven modeling is completely available at https://github.com/TengMichael/battery-health-evaluation.
