# Supplementary material for: Battery health evaluation using a short random segment of constant current charging
Source: iScience. 2022 Apr 12;25(5):104260. doi: 10.1016/j.isci.2022.104260 (PMC9062330; doi:10.1016/j.isci.2022.104260)
Supplement: Document S1. Figures S1–S9 and Table S1 [file mmc1.pdf]

iScience, Volume *25*

Supplemental information

Battery health evaluation using a short random segment of constant current charging

Zhongwei Deng, Xiaosong Hu, Yi Xie, Le Xu, Penghua Li, Xianke Lin, and Xiaolei Bian

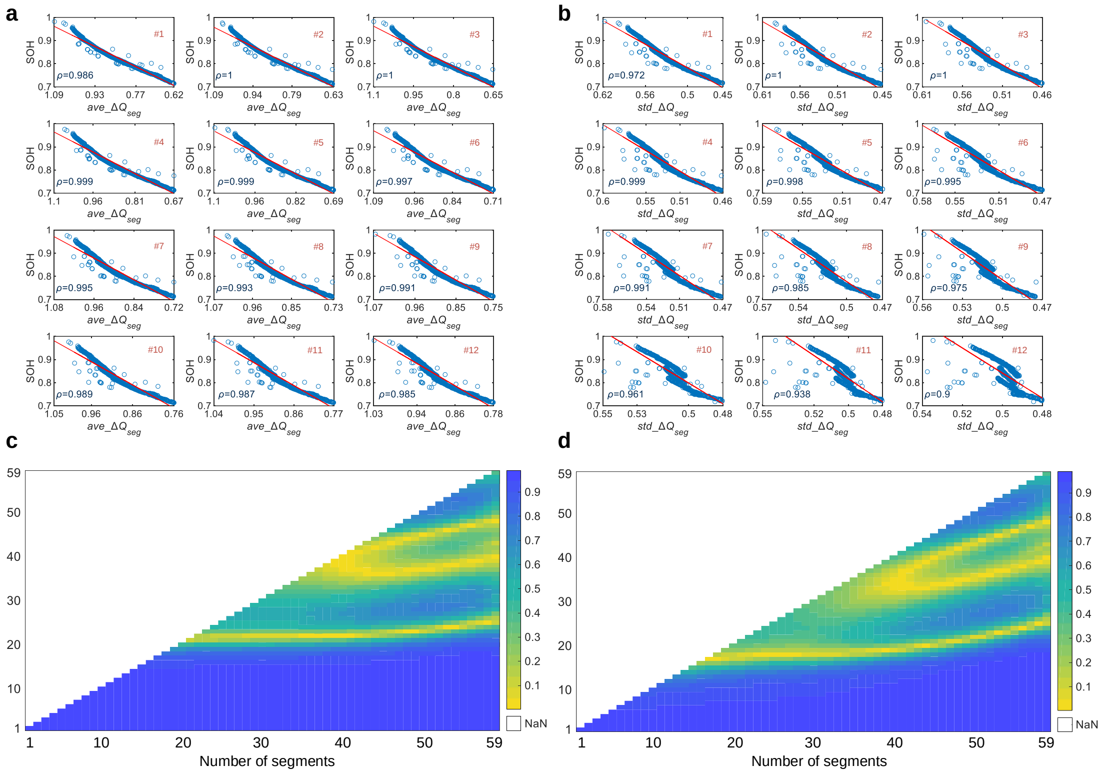

Figure S1. Correlation analysis of extracted features for NCA battery, related to Figure 3. The charge capacity sequence corresponding to the voltage range of 3.60V-4.19V is divided into *m* segments (△***Q****seg*) according to (Equation 2). The *ρ* between the features and battery SOH are analyzed. **a** Correlation of *ave_*△*Q**seg* for each segment when *m* is equal to 12. **b** Correlation of *std_*△*Q**seg* for each segment when *m* is equal to 12. **c** Correlation of *ave_*△*Q**seg* for each segment as *m* varies from 1 to 59. **d** Correlation of *std_*△*Q**seg* for each segment as *m* varies from 1 to 59.

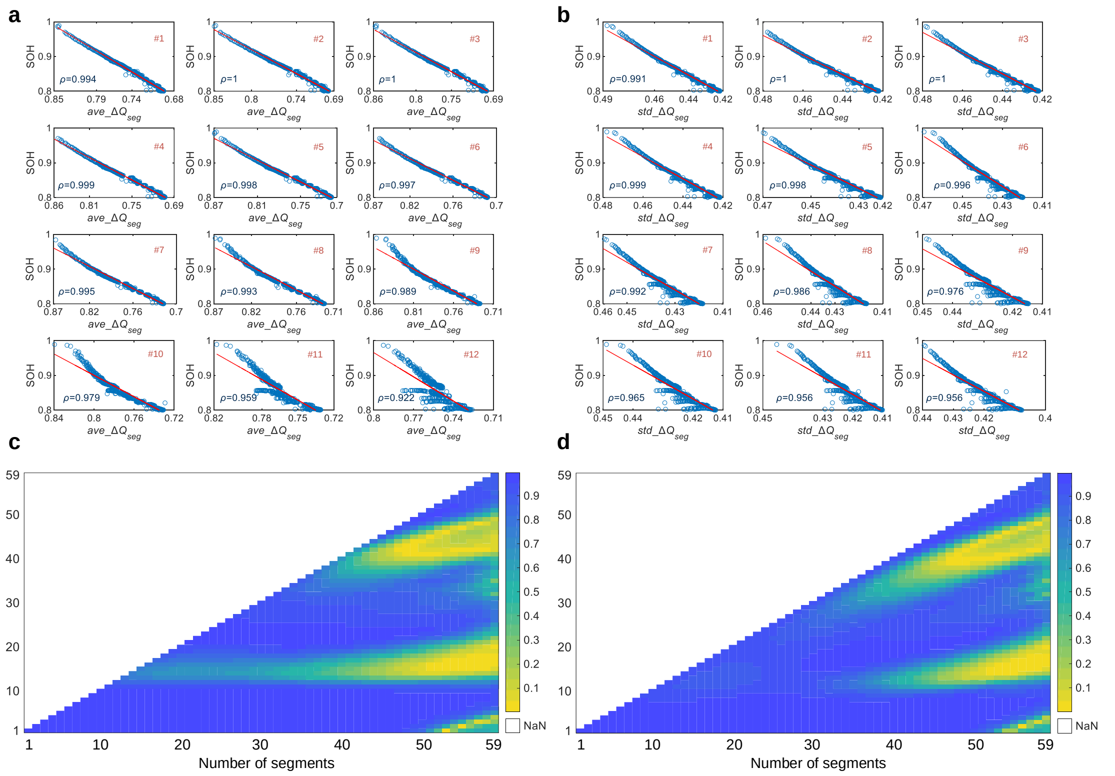

Figure S2. Correlation analysis of extracted features for NMC battery, related to Figure 3. The charge capacity sequence corresponding to the voltage range of 3.60V-4.19V is divided into *m* segments ($\triangle \boldsymbol{Q}_{seg}$) according to (Equation 2). The *ρ* between the features and battery SOH are analyzed. **a** Correlation of $ave\_\triangle Q_{seg}$ for each segment when *m* is equal to 12. **b** Correlation of $std\_\triangle Q_{seg}$ for each segment when *m* is equal to 12. **c** Correlation of $ave\_\triangle Q_{seg}$ for each segment as *m* varies from 1 to 59. **d** Correlation of $std\_\triangle Q_{seg}$ for each segment as *m* varies from 1 to 59.

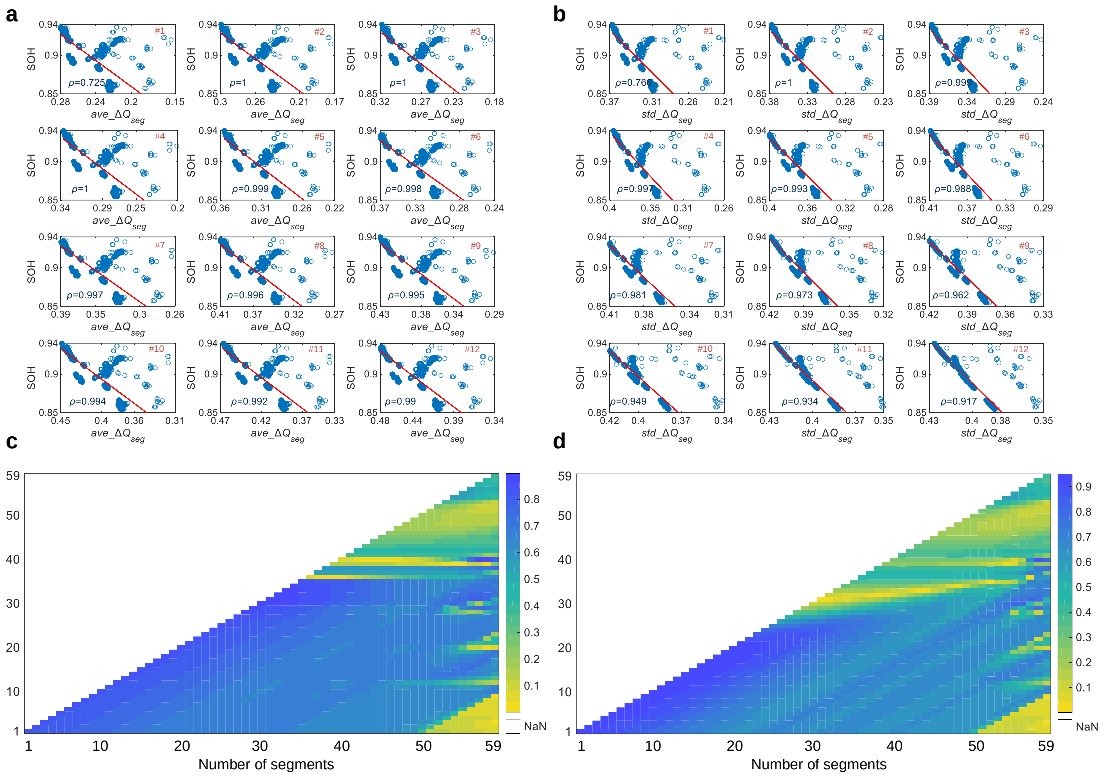

Figure S3. Correlation analysis of extracted features for LFP battery, related to Figure 3. The charge capacity sequence corresponding to the voltage range of 3.00V-3.59V is divided into *m* segments (△$\boldsymbol{Q}_{seg}$) according to (Equation 2). The ρ between the features and battery SOH are analyzed. **a** Correlation of *ave*_△Q_{seg} for each segment when *m* is equal to 12. **b** Correlation of *std*_△Q_{seg} for each segment when *m* is equal to 12. **c** Correlation of *ave*_△Q_{seg} for each segment as *m* varies from 1 to 59. **d** Correlation of *std*_△Q_{seg} for each segment as *m* varies from 1 to 59.

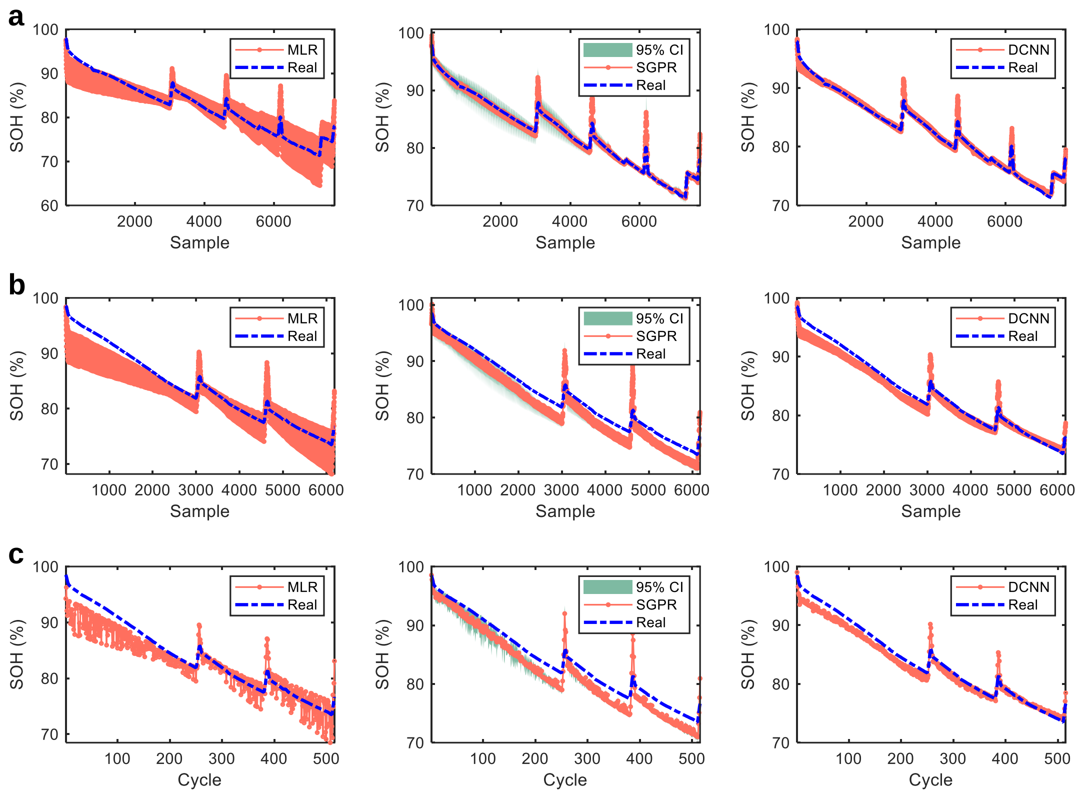

Figure S4. SOH estimation results of NCA batteries, related to Figure 5. The capacity sequence is divided into 12 segments. MLR, SGPR and DCNN methods are used to estimate battery SOH. Dash line represents actual values and the dash dot line represents the estimated values. **a** Training results. **b** Test results using all segments for each cycle. **c** Test results using a random segment for each cycle.

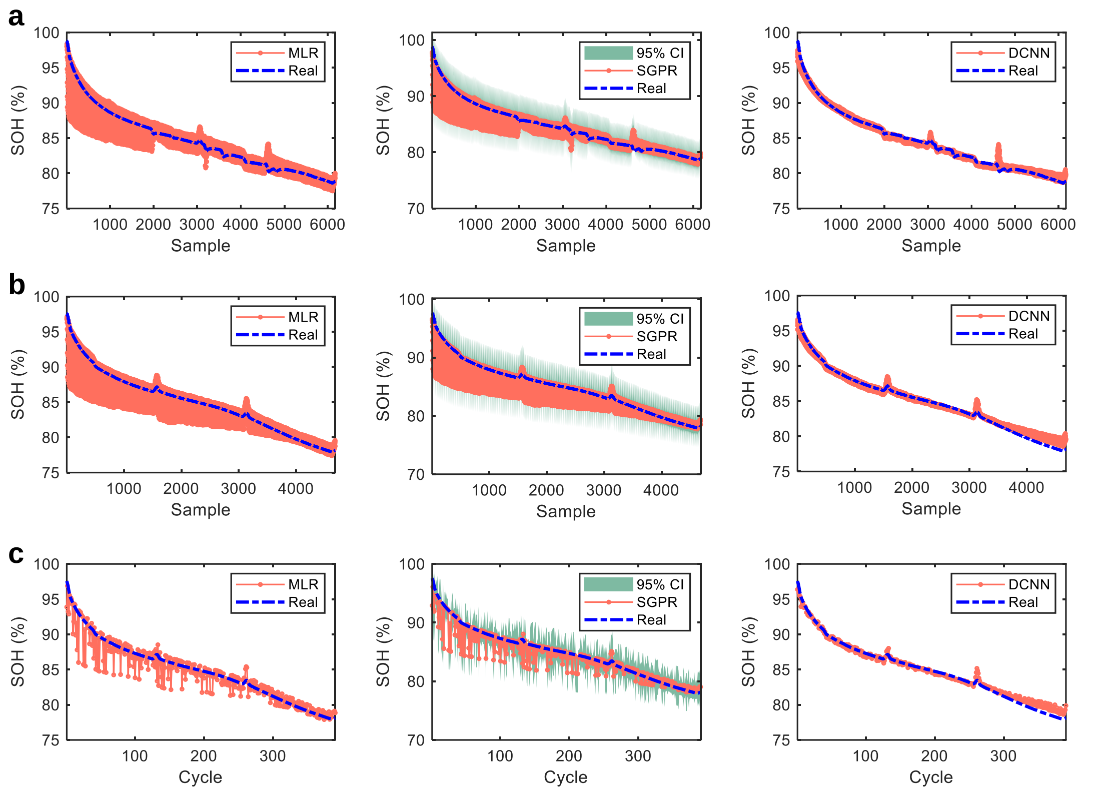

Figure S5. SOH estimation results of NMC batteries, related to Figure 5. The capacity sequence is divided into 12 segments. MLR, SGPR and DCNN methods are used to estimate battery SOH. Dash line represents actual values and the dash dot line represents the estimated values. **a** Training results. **b** Test results using all segments for each cycle. **c** Test results using a random segment for each cycle.

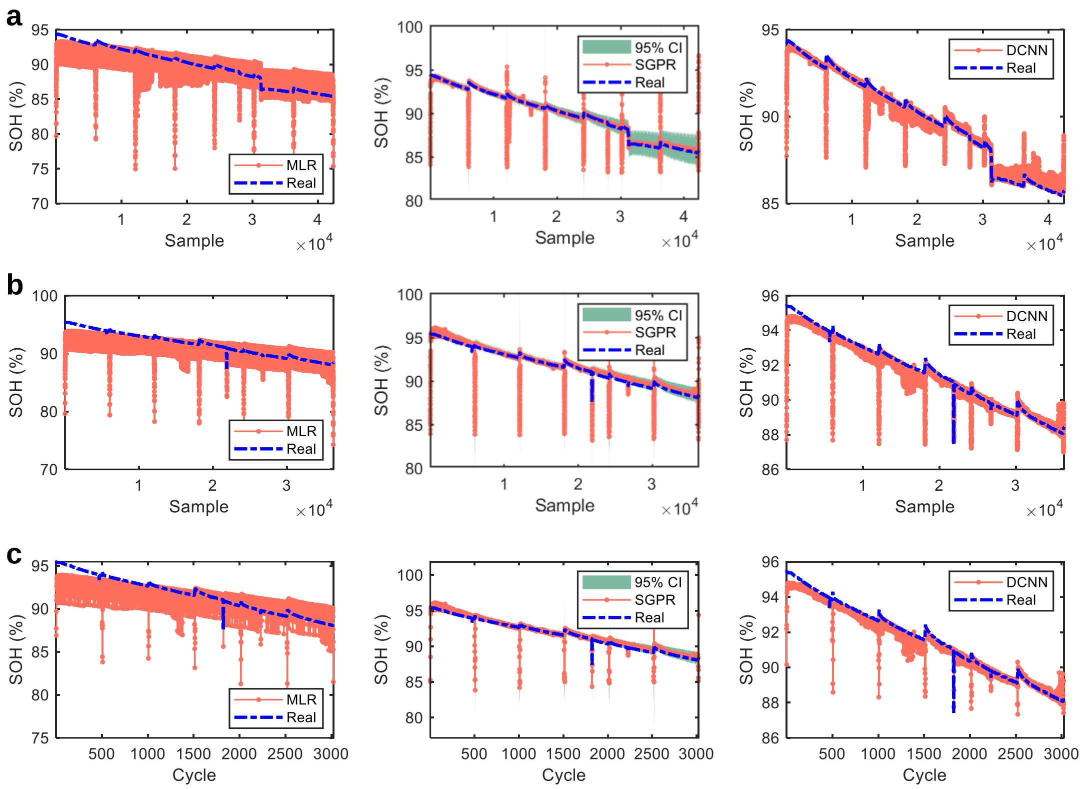

Figure S6. SOH estimation results of LFP batteries, related to Figure 5. The capacity sequence is divided into 12 segments. MLR, SGPR and DCNN methods are used to estimate battery SOH. Dash line represents actual values and the dash dot line represents the estimated values. **a** Training results. **b** Test results using all segments for each cycle. **c** Test results using a random segment for each cycle.

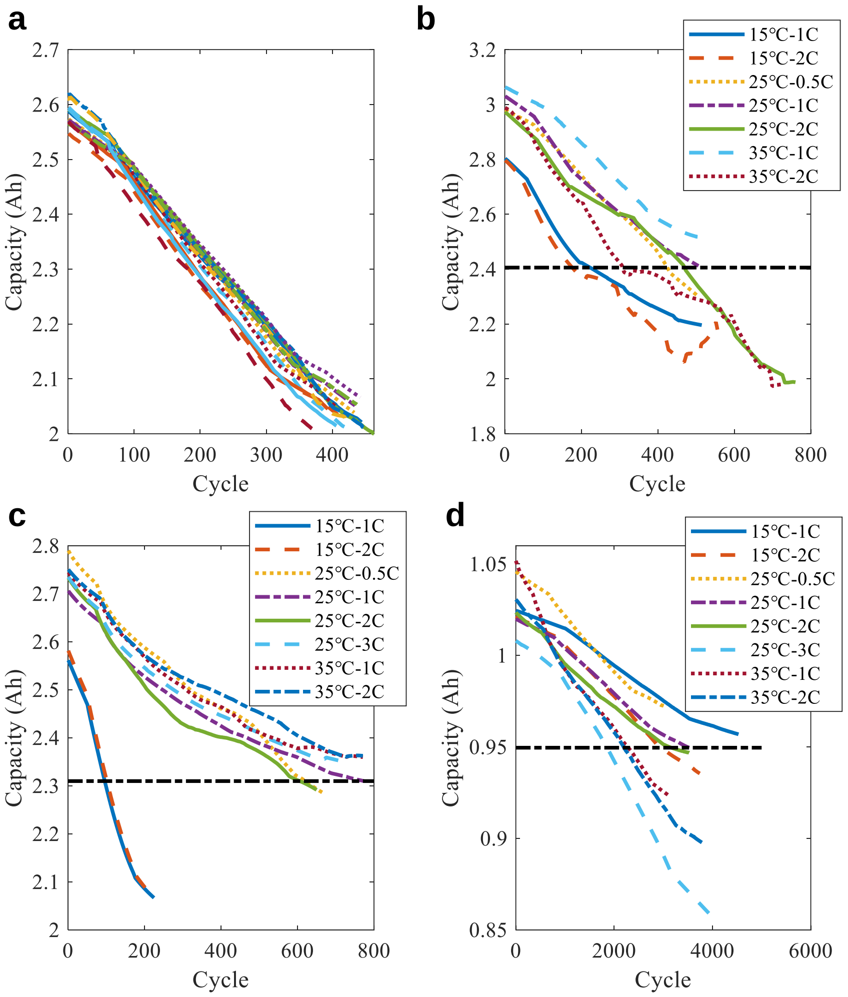

Figure S7. Capacity curves vs cycle number for the four types of batteries, related to Table 2. The symbol “T1-C1” in legends denotes the cell is cycled in a chamber with T1 temperature and C1 discharging rate. The area under the black dash dot line is the capacity range to which the battery under a nominal cycling condition have not decayed. **a** NMC-LCO cells. **b** NCA cells. **c** NMC cells. **d** LFP cells.

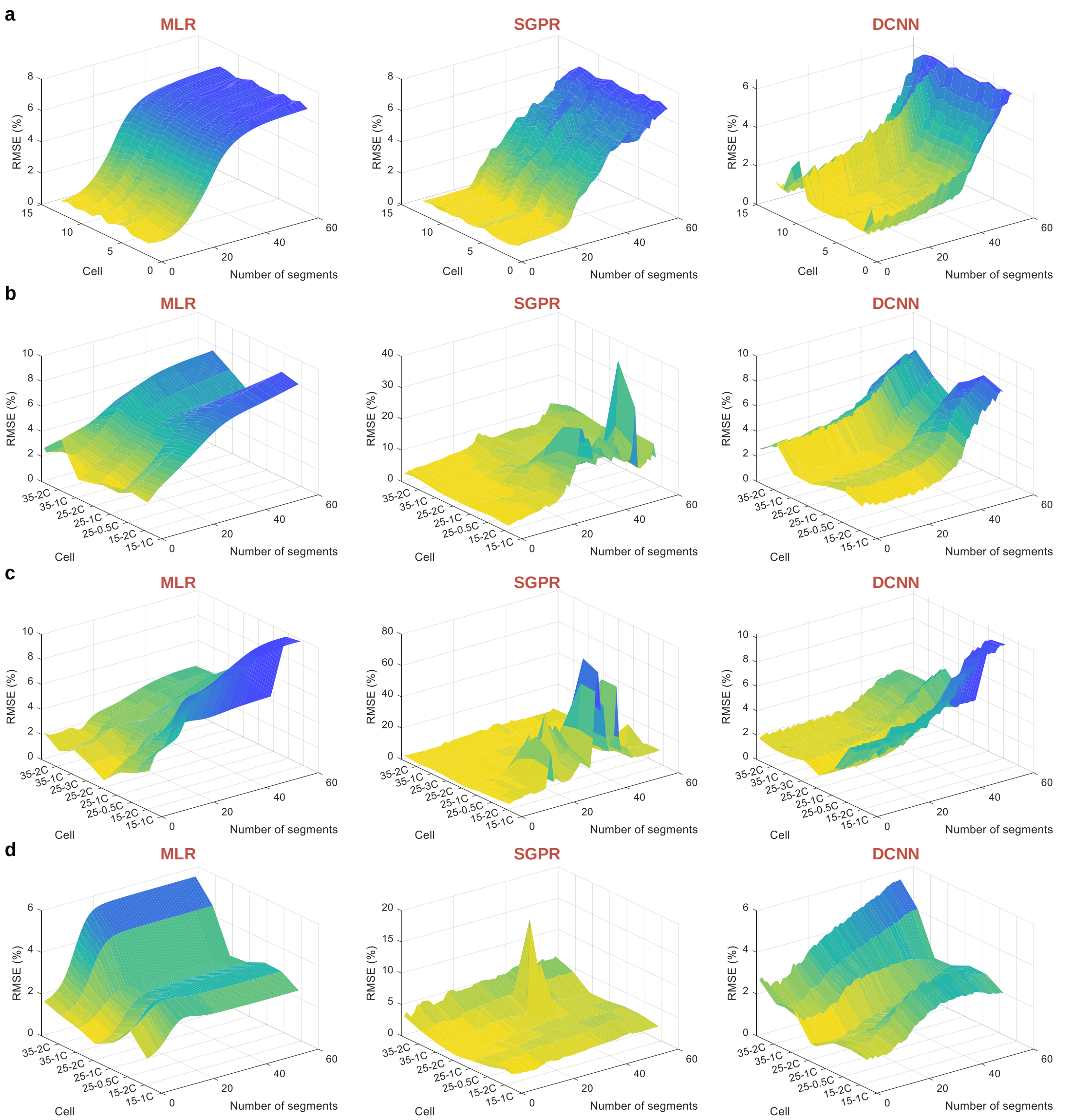

Figure S8. The RMSEs of SOH estimation for four types of batteries, related to Figure 6. Using the data of one cell to train MLR, SGPR and DCNN models, and the remaining cells are used to test the models. The variation of errors with the number of segments is also given. The symbol “T1-C1” in tick labels denotes the cell is cycled in a chamber with T1 temperature and C1 discharging rate. **a** NMC-LCO cells. **b** NCA cells. **c** NMC cells. **d** LFP cells.

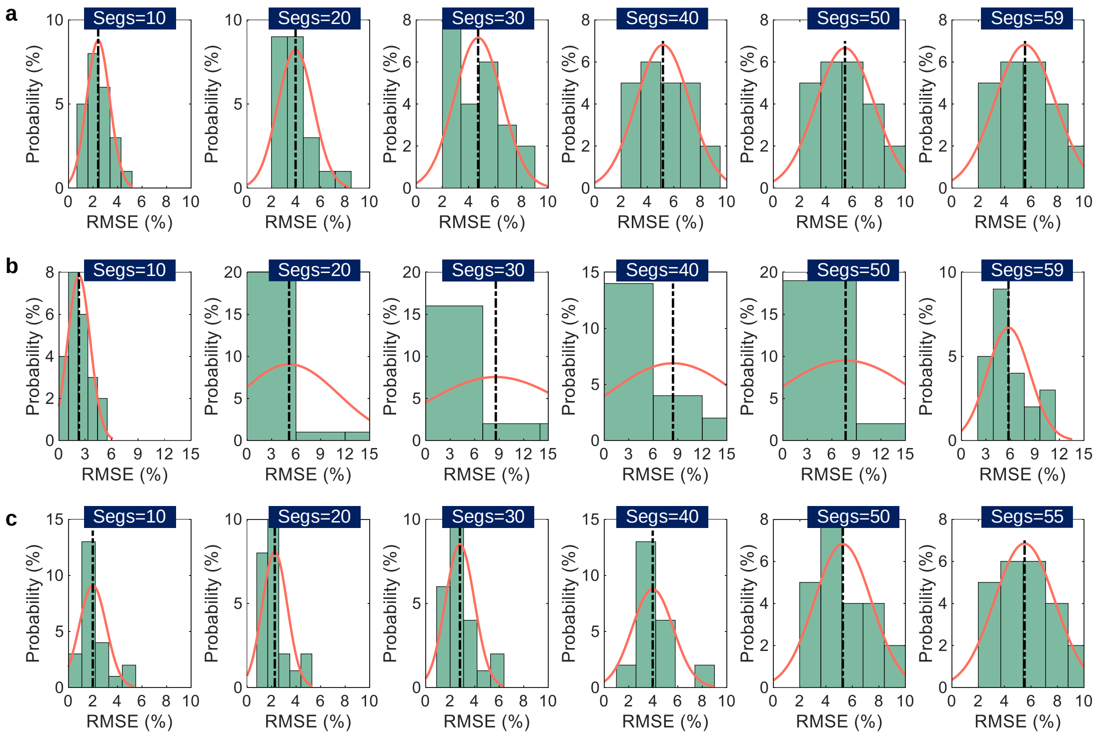

Figure S9. The distributions of RMSEs at different number of segments, related to Figure 7. The vertical dotted line represents the position of mean value, and the symbol "segs" denotes the number of segments. **a** MLR method. **b** SGPR method. **c** DCNN method.

Table S1. The structure of the developed DCNN, related to STAR Methods.

Layers	size	filters
1D convolutional layer	[floor(n/2), 2]	10
1D convolutional layer	[floor(n/2)-2, 2]	10
Maximum pooling layer	[4, 1]	-
Fully-connected layer	1	-
